# Position Control and Force Estimation Method for Surgical Forceps Using SMA Actuators and Sensors

**DOI:** 10.3390/ma14175111

**Published:** 2021-09-06

**Authors:** Dennis Braun, David Weik, Sophia Elsner, Sandra Hunger, Michael Werner, Welf-Guntram Drossel

**Affiliations:** 1 Fraunhofer Institute for Machine Tools and Forming Technology, Department of Medical Engineering, Nöthnitzer Str. 44, 01187 Dresden, Germany; david.weik@iwu.fraunhofer.de (D.W.); sophia.elsner@iwu.fraunhofer.de (S.E.); sandra.hunger@iwu.fraunhofer.de (S.H.); michael.werner@iwu.fraunhofer.de (M.W.); Welf-Guntram.Drossel@iwu.fraunhofer.de (W.-G.D.); 2 Professorship for Adaptronics and Lightweight Design in Production, Chemnitz University of Technology, Straße der Nationen 62, 09111 Chemnitz, Germany

**Keywords:** shape memory alloys, niti, surgical instrument, actuation, force feedback

## Abstract

Minimally invasive surgery is increasingly used in many medical operations because of the benefits for the patients. However, for the surgeons, accessing the situs through a small incision or natural orifice comes with a reduction of the degrees of freedom of the instrument. Due to friction of the mechanical coupling, the haptic feedback lacks sensitivity that could lead to damage of the tissue. The approach of this work to overcome these problems is to develop a control concept for position control and force estimation with shape memory alloys (SMA) which could offer haptic feedback in a novel handheld instrument. The concept aims to bridge the gap between manually actuated laparoscopic instruments and surgical robots. Nickel-titanium shape memory alloys are used for actuation because of their high specific energy density. The work includes the manufacturing of a functional model as a proof of concept comprising the development of a suitable forceps mechanism and electronic circuit for position control and gripping force measurement, as well as designing an ergonomic user interface with haptic force feedback.

## 1. Introduction

### 1.1. State of the Art

Minimally invasive surgery (MIS) is characterized by using long, narrow instruments to operate through natural orifices or small incisions [1]. In the beginning, the technique was mainly used for diagnostic purposes in gynaecology and laparoscopy [2]. During the twentieth century, accompanied by technical advancements in endoscopy, it is increasingly used for treatment in various medical fields such as otorhinolaryngology and neurosurgery [3]. Patients benefit from experiencing less pain due to the reduction of trauma of the tissue, which leads to a shorter period of convalescence [4,5]. Although cost for instruments and durations of surgical procedures are increased compared to open surgery, the shorter period of hospitalization can reduce the total cost for MIS [6]. However, for the surgeons, many disadvantages result from the surgical requirements and the design of currently availably instruments. Problems arise from a lack of degrees of freedom, scaling of hand and tip forces, mirroring of movements and absence of direct sight [7]. Depending on the task, the instrument’s low mechanical efficiency with tendons can decrease performance and sensitivity of the haptic feedback [8,9]. Uncomfortable holding positions and bad ergonomics are the reasons why most surgeons have physical symptoms due to MIS [10]. Digitalization is already taking place in the development of surgical instruments and there is an increased use of robotic surgery. During this transformation, away from highly complex mechanically actuated instruments, not all requirements have already been met by their prospective successors. There has been research on the development of a manually actuated laparoscopic instrument with haptic feedback, which is promising but lacks the possibility of functions like force scaling or automated operation. [11]. Robotic systems such as the Da Vinci (Intuitive Surgical, Inc.) provide good ergonomics, yet insufficient haptic feedback and increase costs of general surgery procedures for the health system compared to laparoscopically performed surgery [12,13]. To our knowledge, handheld actuator-driven laparoscopic instruments offering haptic feedback do not yet exist, but could provide a beneficial combination of advantages of the available systems.

### 1.2. Load Sensing and Haptic Feedback

Conventional MIS instruments use mechanical couplings like tendons or pull rods for actuation. In such instruments, mechanical friction occurs by design and is a problem for surgeons, because kinesthetic (force) and cutaneous (tactile) feedback are reduced or eliminated [14]. In actuator based instruments, the lack of haptic feedback occurs due to the abolishment of a direct mechanical linkage. While many surgeons claim to learn how to work with visual instead of haptic feedback [15], other studies state that MIS with some sort of haptic feedback is beneficial [16,17]. Establishing haptic feedback requires the implementation of adequate sensors to measure forces directly on the effector, which is a major technical challenge [14]. The sensors need to be isolated against temperatures, sealed against liquids and have minimal dimensions. Former research mainly focused on the integration of load sensing in instruments for robotic surgery [18,19,20]. In laparoscopic instruments however, innovation in haptic feedback seems to be neglected, even though the need is clearly stated. In a study in 2009, 79% of all asked laparoscopic surgeons working in European hospitals “maintain that it is necessary to have a new laparoscopic grasper with augmented feedback” and 77% “would like to have tactile feedback as an indication of the level of pinch force” [21].

### 1.3. Shape Memory Alloys

**Figure 1:**
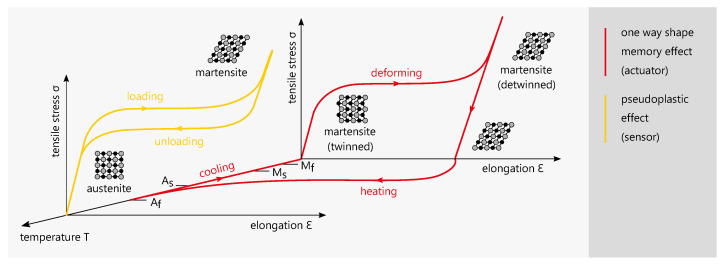
Examplary stress-elongation-temperature diagrams the one way shape memory effect (OWSME) and the pseudoelastic effect (PE) [26,27].

Shape memory alloys (SMA) are sometimes referred to as smart materials due to their unique material properties [22]. The most common SMA is nickel-titanium (NiTi), which has a high biocompatibility and is therefore used in many medical applications such as dental braces and stents [23]. Because of the small usable space inside of an instrument, actuation elements need to deliver a high force output while having a minimal footprint. SMA actuators have the highest energy density compared to other actuation technologies [24,25] and are therefore suitable for this application. Additionally, NiTi elements can be used as elongation sensors. Figure 1 shows examplary stress-elongation-temperature diagrams for the one way shape memory effect (OWSME) and the pseudoelastic effect (PE).

The NiTi alloys used for actuation and sensing differ in their elemental ratio and the resulting transformation temperatures. For actuation, the OWSME is used and the transformation temperatures are above ambient temperature. When the actuator is heated, the microstructure changes from a martensitic to an austenitic phase and the actuator ‘remembers’ its original shape. During cooling, the material will retain the shape while the microstructure becomes martensitic (twinned). From this state, it can be mechanically deformed again (detwinned) to be operated cyclically. The transformation temperature indices $M_{s}$, $M_{f}$, $A_{s}$ and $A_{f}$ signify the start or finish of the transformation to a complete austenite oder martensite structure of the specific alloy. The PE occurs when temperatures are above austenite finish temperature $A_{f}$. By mechanical loading, a microstructural change from austenite to martensite is induced. The characteristic stress plateau is reversibly cycled with a hysteresis and no temperature change is required. Due to the correlation of the electrical resistance change ΔR/R and the elongation ε the effect can be used for elongation sensing.

## 2. Materials and Methods

### 2.1. Concept and Approach

**Figure 2:**
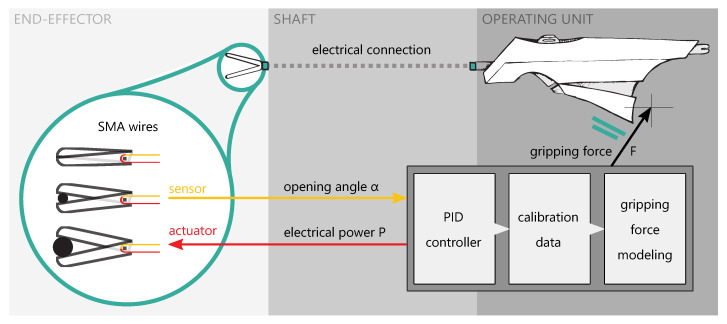
Schematic overview of the instrument model: Loads applied to the forceps are sensed by a modeling method coupled to the PID position control and a priori collected calibration data and are transferred to the operating unit as an electric signal.

The goal of this work is to develop a method of using SMA elements as actuators and sensors to establish position control and force estimation which could offer advanced functionality to instruments for the MIS. As a proof of concept, a functional model of such an instrument is manufactured and evaluated. Figure 2 shows an overview of the instrument model, which consists of an operating unit and an end-effector, connected by a shaft.

The signal flow between the components is only electrical. The connection can therefore be seen as a master-slave-system with the shaft only providing a mechanical fixation for the parts. This allows for user specific gripping force scenarios, gripping force limitation, haptic feedback and other features. As a result, the instrument can be tuned adaptively to meet the requirements of the surgical procedure and the surgeon’s personal preferences. This research paper focuses on development of the end effector in the form of forceps and control method to measure gripping forces which are then transfered and generated as feedback to the operating unit. Our approach differs from existing methods because loads applied to the forceps are sensed by a modeling method coupled to the position control. Other works related to load sensing forceps with SMA elements require additional load cells [28] or DC-motors [29]. In our concept, no extra parts are needed but the SMA wires to actuate and measure the forceps opening. Therefore, this principle can potentially be miniaturized and capsulated to fit into the dimensional space of an instrument and guarantee thermal and electric insulation. With the electronic-based control, the tactile feedback is not directly coupled to the mechanical friction, as it is generated by a separate feedback module controlled by software.

### 2.2. NiTi as Actuator and Sensor

Nickel-titanium alloys are commercially available mainly as raw stock sheet metal, wire or tube. Wire is the most common form and the easiest to work with, because elongation and actuation forces are generated axially. SMA actuator wires contract on activation and therefore generate a pulling force $F_{A}$, which can be calculated by multiplying the maximum tensile stress of the alloy $\sigma_{PS}$ by the effective wire cross-section $A_{CS}$:

(1) $$\sigma_{PS}=\frac{F_{A}}{A_{CS}}\;\to \;F_{A}=\sigma_{PS}\;\cdot \;\pi \;\cdot \;\frac{d^{2}}{4}.$$

A wire diameter of 0.3 mm is used for the actuator wire, which can generate a force of up to 35 N. Heating of the wire can be realized electrically by resistance heating. For the OWSME to be operated cyclically, an additional force is needed to restore the initial state by elongating the actuator wire. If a spring is used for resetting, the spring force has to be subtracted from the effective actuator force, which results in a loss of efficiency. This is not the case in an antagonistic arrangement of two actuator wires. Research shows the potential of the antagonistic approach to be able to operate fast and accurate [30,31]. An actuator wire can only be resetted when cooled down below martensite finish temperature $M_{f}$. To ensure cooling, $M_{f}$ should be above the ambient temperature, in this case body temperature. A maximum tensile stress $\sigma_{PS}$ of 500 MPa was defined for *n* of 1000 life cycles according to Langbein et al. [32]. The sensor wire is also made from NiTi, but has different transformation temperatures. To use the PE and guarantee a full austenite structure when no load is applied, $A_{f}$ has to be below body temperature. The sensor wire is a passive element that is not activated by heat, but pre-tensioned mechanically. To keep the spring force low, the sensor wire diameter is chosen to be very small at 0.05 mm. The used wires are characterized in Table 1.

### 2.3. Effector Mechanics

**Figure 3:**
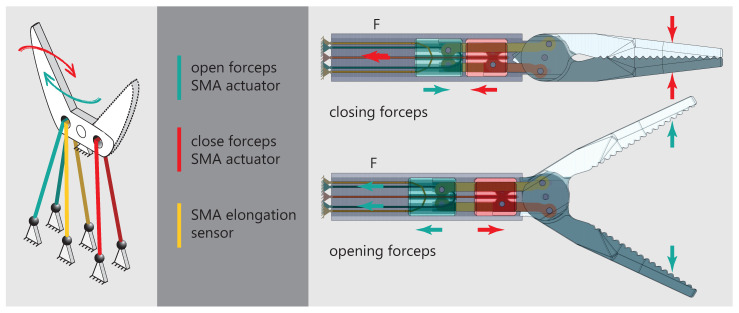
(**Left**): Graphic representation of the seesaw mechanism and the arrangement of actuators and sensors. (**Right**): Final mechanism design utilizing two cylinders to synchronize the movement of both forceps brackets.

Effectors used on instruments for the MIS are usually different kinds of forceps, grippers, scissors and needle holders [33]. Manually actuated instruments utilizing push and pull rods are the state of the art. Because of the way how SMA actuators work, a new mechanical design has to be developed. The documented gripping forces during laparoscopic surgeries are in a wide range depending on the specific procedure. Okuda et. al recently found out that structural damage of liver tissue is observed at gripping forces of 2.5 N [34]. A gripping force study on an animal model shows similar values [35]. In several dissection tasks using an instrument with force feedback, the gripping force never exceeded 5 N [36]. The challenge in designing the mechanism is to achieve high gripping forces and quick movements at the same time. The pulling force generated by the wire actuator can be turned into a rotation of the forceps by applying the basic principle of the lever. In an antagonistic arrangement of two actuator wires, the mechanism is similar to a seesaw. Figure 3 displays the basic principle (left) and the final mechanism design (right).

Both wires can be deflected around the forceps mounts, to gain even higher gripping forces. As a result, the pulling force doubles and connectivity is simplified, because both ends of the wire are on the proximal side of the instrument. Compared to one wire with the same pulling force, the surface-area-to-volume ratio is increased by using two smaller wires which increases cooling performance. In the final principle, a sensor wire is added on the side of the opening actuator. Because the sensor acts as a tension spring, the forceps will be open in a non-powered state and prevent tissue damage. To synchronize the movements of both forceps brackets, additional levers connect the opening lever mounts as well as the closing lever mounts of each bracket. The levers mount to cylinders which act as linear guides inside the main tube of the instrument. The actuator for closing the forceps (red) is mounted to the red cylinder, the actuator for opening the forceps (green) and the sensor wire (yellow) are mounted to the green cylinder. Exemplary for the forceps closing, the red actuator wire contracts and pulls back the red cylinder to which it is connected. Because of the red lever connecting the red cylinder to the forceps, the forceps rotates in the closing direction. At the same time, because of the way the yellow lever connects the forceps to the green cylinder, the green cylinder moves in the distal direction of the instrument and therefore stretches the green actuator wire. This new mechanism design makes it possible to realize both movement directions of the forceps by pulling forces of the respective actuator. In addition, the design ensures cyclic resetting by elongating one actuator when its antagonist is contracting.

### 2.4. Control and Electronics

**Figure 4:**
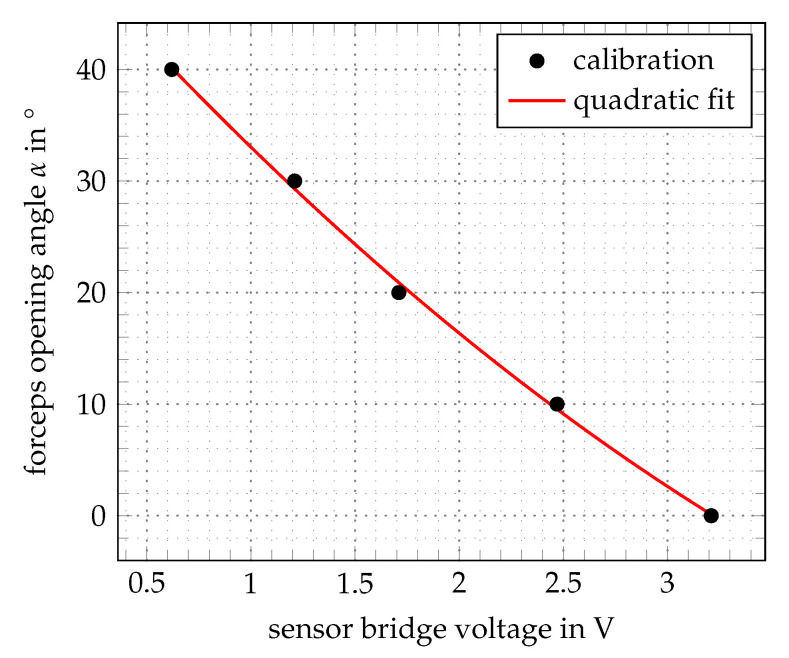
Calibration of the measured sensor signals to the opening angle.

The developed control method is based on a relative-model that uses calibrating parameters as ground truth obtained from a specific assembly, instead of quantified SMA modelling [37]. The electric resistance of NiTi wires depends on physical parameters such as temperature, mechanical stress, elongation and microstructure. One can not differentiate between these influences when measuring the resistance of an actuator wire, that is activated by heat and affected by altering mechanical stresses [32]. For this reason, an additional sensor wire is integrated, which has pseudoelastic properties (austenitic phase) and acts a passive element. The sensor is used as a variable resistor depending on the effective mechanical strain. Integrated into the forceps, the sensor wire is elongated from its preloaded state when the forceps close and therefore measures the opening angle. Within the electronic design, the relative measurement of the sensor resistance is realized with a bridge circuit and an instrumentational amplifier. A calibration is done that refers the sensor bridge voltage to the opening angle of the forceps, as shown in Figure 4. The transfer function is fitted with a quadratic polynomial to measure the opening angle α, which is used for the control of the forceps.

As control design, the well known proportional-integral-derivative (PID) controller approach is used [38]. The coefficients $K_{p}$, $K_{i}$ and $K_{d}$ are the proportional, integral and derivative terms in the control function:

(2) $$u\left(t\right)=K_{p}\;e\left(t\right)+K_{i}\int_{0}^{t}e\left(\tau \right)\;d\tau +K_{d}\;\frac{de(t)}{dt}.$$

**Figure 5:**
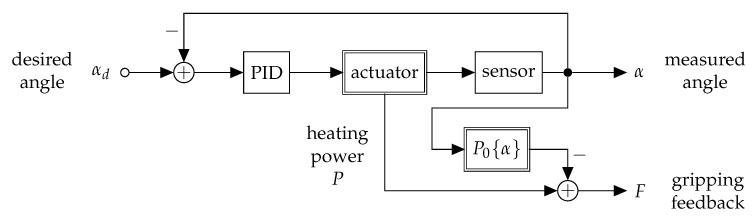
Signal flow chart for the control of the forceps opening angle and the gripping force feedback estimation. The actuator module is comprised of both actuation wires, which are excited by either positive or negative PWM-signals from the PID-controller. Double frame blocks indicate elements with a non-linear transfer function. The transfer functions of the sensor and the no-load power $P_{0}$ have to be quantified in an a-priori calibration.

The term u(t) represents the control voltage for heating the actuator, *e* is the control error calculated as the difference between the desired setpoint (set by the user via potentiometer) and the actual forceps angle (measured by the sensor wire). The controller and the signal processing are implemented in a microcontroller based on the Atmel ATmega328 via the PID_V1.h library. The microcontroller is attached onto a developed electronic board that contains 5 V MOSFET power switches, current and voltage sensors for each actuator and the sensor bridge. The difference or rather the error signal between the desired and measured angle is filtered with a 4 Hz low-pass and fed into the PID, which generates a pulse-width-modulated (PWM) signal [39]. For negative differences, the forceps closing actuator is activated and for positive differences the opening actuator. The setup is shown in Figure 5.

To guarantee cooling and reduce the mechanical stress of the actuators, a delay of 0.5 s is implemented for the transition between opening and closing. The feedback signal is also generated by using a relative-model and an a priori calibration. In an idle run, where external forces are applied to the forceps, the device is controlled to remain in several opening angles and the associated holding power $P_{0}$ is measured. This value is subtracted from the heating power *P* to generate the feedback signal *F*. This approach supposes that the heating power exceeds the idle run at a certain opening angle α, if an object is grasped in the forceps. The difference in amplitude gives an estimate of the gripping force. The feedback signal is filtered with a 0.5 Hz low-pass to suppress high initial heating powers and is transferred to the operating unit. The literature describes haptic feedback modules using SMA for actuation [40,41,42]. However, the design and development of the haptic feedback module is not a focus of this work and the demonstration is realized via a vibration motor, that vibrates with short pulses that have less delay depending on the amplitude of the sensed gripping force.

### 2.5. Functional Model Manufacturing

**Figure 6:**
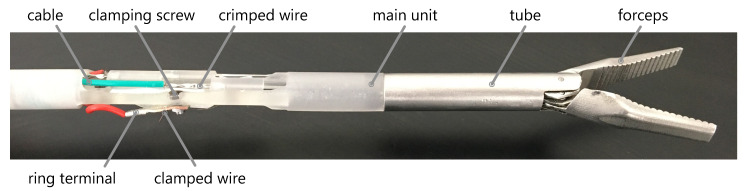
Functional module of the effector module manufactured by SLM, STL and EDM technologies.

The end-effector module and the operating unit are manufactured in various materials using rapid prototyping technologies. The functional model of the effector module is shown in Figure 6.

Selective laser melting (SLM) was used to produce the forceps brackets in stainless steel (1.4404) to ensure the required rigidity (Truprint 1000, TRUMPF GmbH + Co. KG). The process parameters are listed in Table 2.

**Figure 7:**
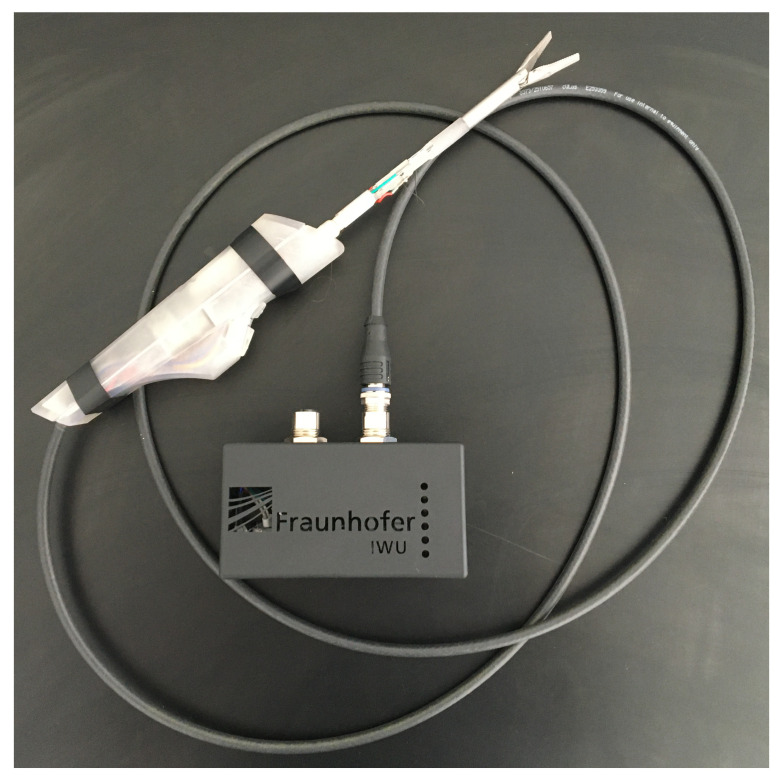
Functional model consisting of effector module, operating unit and electronics box.

The tube to which the forceps mount to and the levers pulling the forceps are also made from stainless steel (1.4301) using conventional milling as well as continuous wire electrical discharge machining (EDM). Other parts are made from thermoplastic resin (Clear Resin, Formlabs GmbH) using stereolithography (STL) on a Formlabs 2 machine. This technology is beneficial, because it provides maximum design freedom and the material is electrically insulating. One end of every actuator and sensor wire is crimped using a stainless steel tube that also holds the copper cable. The crimp sits in a recess inside the main part, just like a Bowden cable. The other end of every wire is clamped by a screw, together with a ring clamped terminal connected to the copper cable. This way, the effective length and therefore the preload of the wire can be adjusted. Resetting the wires had to be done after the first few cycles by preloading of 80 MPa, which translates to a pulling force of 6 N [32]. A ten pin micro medical connector (Nextronics Engineering Corp.) is utilized to connect the shaft of the effector module to the operating unit. The developed electronics board is fit into a box utilizing two GX16 sockets to connect instruments to, as well as the power supply. Figure 7 shows the manufactured functional model.

Six LEDs on the box display a visual feedback of the gripping force, with more LEDs lighting up on increasing forces. The operating unit is designed to provide good ergonomics when held below the waist as it’s usual for laparoscopic procedures. It features a linear potentiometer to control the opening angle of the instruments with the thumb. Buttons for additional features like steering can be placed at the bottom, to be reached with index and middle finger.

## 3. Results and Discussion

**Figure 8:**
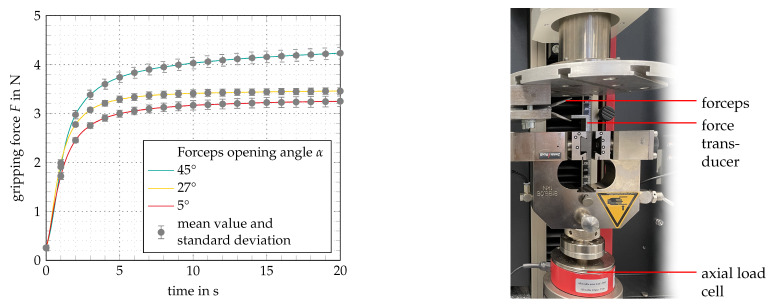
Gripping force measurement at opening angles α of 5°, 27° and 45° over a time of 20 s and 10 runs. (**Left**): Diagram showing mean values and standard deviations. (**Right**): Measurement setup at opening angle α of 45°.

During the evaluation, the functional model is tested by measuring its technical specifications in a regular use scenario in which various objects are gripped. Regarding the dynamics, complete closing as well as opening the forceps takes about one second, which is slower than what is possible on manually actuated instruments, but considered sufficient by surgeons interviewed in the development process. The position control gives an accurate feel, the opening angle can be adjusted even in small movements and any desired opening angle can be accomplished. When the direction is changed between opening and closing, a subtle delay can be perceived, which derives from the programmed delay needed to cool the antagonistic wire. In the prototyping state, feedback of the gripping force is realized haptically using a vibration motor and visualized with an LED on the tip of the operating unit. When an object is gripped, the haptic feedback is activated after a delay of about one second, which involves the risk of damaging tissue meanwhile. To measure the maximum gripping forces, a 100 N axial load cell is used in a testing machine (ZwickiLine, ZwickRoell GmbH Co. KG). Figure 8 shows the measuring setup as well as the diagram of mean value and standard deviation of the gripping force measured during ten runs at forceps opening angles of 5°, 27° and 45°.

As the diagram shows, the maximum gripping force *F* increases with rising opening angles α, which firstly is due to the kinematic properties of the mechanism. Secondly, this effect occurs because of SMA actuator characteristics. At higher elongations (meaning larger opening angles) the effective mechanical loads are higher. The maximum gripping force *F* of 4.23 N ± 0.13 N was measured at an opening angle α of 45°. Similar works related to SMA actuated surgical grippers documented maximum gripping forces of 0.9 N [43] to 5.5 N [44]. As stated before, much higher gripping forces are not desirable due to the risk of tissue damage, so this is an adequate value. However, gripping forces only increase slowly. After two seconds, the gripping force *F* at an opening angle α of 45° is 2.98 N ± 0.09 N. Altogether the gripping force requirement has been met in the functional model. Shortcomings are related to the general latency of the system, which has to be further optimized. The achieved specifications are listed in Table 3.

## 4. Conclusions

This work adressed the need for haptic feedback in handheld electrically actuated instruments for MIS. SMA elements were considered as an adequate resource because of the high work capacity of the shape memory effect as well as their capability to sense elongation in the superelastic phase. Driven by this, a method to estimate the gripping force could be established with a PID position control model and calibration. The main results and novelties of this work are:

- The approach to realize a software control model to use position control for gripping force estimation instead of using external load sensors is a novelty. The developed control model is not based on a physical-oriented model but rather a relative-model on calibrating parameters as ground truth within a specific assembly.
- A new forceps mechanism was designed to meet the special requirements of the SMA actuators and sensor. By using two deflected actuators in an antagonistic arrangement, the cooling performance and force output of the forceps could be increased. Another SMA wire acts as an elongation sensor to detect the opening angle of the forceps.
- By mainly using rapid prototyping technologies, a functional model was manufactured. In an evaluation setting, the working principle could be proven. Gripping forces of up to 4.23 N ± 0.13 N could be measured.

The benefits of the approach are:

- Compared to conventional laparoscopic instruments, no mechanical coupling is used. The force transmission between the operating unit and the forceps is adjustable and there is a great freedom in designing the control elements, because the connection is only via an electric signal.
- Compared to robotic systems, it is less complex and therefore more cost-effective.
- By not using an external load sensor, the system can be capsulated, which is important for the cleaning process of the instrument and the prevention of cross-contamination.

Current drawbacks of the functioning model are:

- Slow dynamics of the functioning model can be improved by optimizing the cooling performance and tuning of the PID coefficients.
- The generation of the feedback force is realized via a vibration motor in the functional model. This can be improved to increase usability.
- Thermal management and electric isolation has not yet been addressed, but is planned for future works.

In an upcoming cooperation with surgeons we aim to adapt the principle to their needs in regards to ergonomics and handling. By that, the developed system can make use of its full potential. Due to the actuation and feedback being controlled and generated by software, there are unlimited design possibilities regarding operating concepts. New useful functions such as tissue characterization can potentially be implemented as well.

## Figures and Tables

**Table 1:** Specifications of used NiTi wires as actuators and sensors.

Description	Symbol	Actuator Wire	Sensor Wire
Material		NiTi (Memry Alloy H)	NiTi (Memry Alloy S)
Wire Diameter	*d*	0.3 mm	0.05 mm
Martensite start transformation temperature at zero stress level	$M_{s}$	61.5 °C	14.8 °C
Martensite finish transformation temperature at zero stress level	$M_{f}$	52.8 °C	−12.7 °C
Austenite start transformation temperature at zero stress level	$A_{s}$	72.4 °C	−13.0 °C
Austenite finish transformation temperature at zero stress level	$A_{f}$	85.5 °C	18.3 °C
Specified life cycles of actuator wires	*n*	1000	1000
Maximum tensile stress for *n*	$\sigma_{PS}$	500 MPa	800 MPa
Maximum reversal elongation at $\sigma_{PS}$	$\varepsilon_{PF}$	4.5%	5%

**Table 2:** Forceps brackets manufacturing process parameters with SLS technology on the (Truprint 1000, TRUMPF GmbH + Co. KG) in 1.4404 stainless steel.

	Support Structure	Contour	Area
Laser power $P_{L}$	100 W	75 W	113 W
Velocity $v_{L}$	0.5m/s	0.5m/s	0.7m/s
Spot size $d_{0}$	55 μm	55 μm	55 μm
Layer thickness $T_{L}$	20 μm	20 μm	20 μm

**Table 3:** Technical specifications of the functional model.

Description	Symbol	Value
Instrument diameter	*d*	8 mm
Maximum forceps opening angle α	α	60°
Maximum gripping force at α= 5°	*F*	3.25N ± 0.11 N
Maximum gripping force at α= 27°	*F*	3.45N ± 0.07 N
Maximum gripping force at α= 45°	*F*	4.23N ± 0.13 N
Duration for complete opening/ closing	$t_{C}$	1 s

## Data Availability

Not applicable.
